# Induction of Apoptosis of 2,4′,6-Trihydroxybenzophenone in HT-29 Colon Carcinoma Cell Line

**DOI:** 10.1155/2014/468157

**Published:** 2014-01-22

**Authors:** Ma Ma Lay, Saiful Anuar Karsani, Sri Nurestri Abd Malek

**Affiliations:** ^1^Institute of Biological Sciences, Faculty of Science, University of Malaya, 50603 Kuala Lumpur, Malaysia; ^2^University of Malaya Centre for Proteomics Research (UMCPR), University of Malaya, 50603 Kuala Lumpur, Malaysia

## Abstract

2,4′,6-Trihydroxy-4-methoxybenzophenone was isolated from the ethyl acetate fraction of *Phaleria macrocarpa* (Scheff.) Boerl. fruits. It was found to inhibit cell proliferation in HT-29 human colon carcinoma cell line but caused little damage to WRL-68 normal human liver and MRC-5 normal human fibroblast lung cell lines. The compound was found to sharply affect the viability of HT-29 cells in a dose- and time-dependent manner. HT-29 cells treated with the compound showed morphological changes under microscopic examination such as cell shrinkage, membrane blebbing, DNA fragmentation, and the occurrence of apoptotic nuclei. The percentage of early apoptotic, late apoptotic, and dead or necrotic cells was determined by flow cytometry using annexin V-FTIC/PI staining. In addition, flow cytometry showed that, when the HT-29 cells were treated with 115 *µ*M of the compound, it resulted in G_0_/G_1_ phase arrest in a time-dependent manner. Western blot revealed an upregulation of PUMA, Bak, Bcl-2, and Mcl-1 proteins suggesting that the compound induced apoptosis in HT-29 cells by regulating these proteins.

## 1. Introduction

Cancer refers to a family of diseases in which tissues multiply and spread unregulated throughout the body, the result of which may lead to death [1]. It is a major public health problem and has significantly increased the worldwide death rate. One of the most common types of cancer is colon cancer. Modern advances in cellular and molecular biology have increased our understanding of the various mechanisms of cancer [2]. Nowadays, there are many natural compounds and mechanism-based approaches to cancer treatment that have the potential to be successful. The development of anticancer drugs has also seen a massive contribution from natural products [2].

*Phaleria macrocarpa* (Scheff.) Boerl. is a medicinal plant belonging to the Thymelaeaceae family and in Indonesia it is locally known as Mahkota Dewa. The edible fruits of this plant are usually mixed with other Indonesian herbs and used in herbal medicine [3]. The fruits of *P. macrocarpa* consist of the skin which have a bright red color, a white, fibrous, and watery flesh, a shell, and a seed. In alternative medicine, it is used in the treatment of cancer, diabetes mellitus, nerve pain, kidney failure and disorder, liver dysfunction, serious illnesses, hypertension, skin diseases, and management of cholesterol levels.

Research on the use of the extracts of *P. macrocarpa* fruits as a possible anticancer agent is extensive. Prior research has shown that an ethanol extract from the flesh of the *P. macrocarpa* fruit toxic towards HeLa cell line derived from cervical carcinoma [4]. It has been shown that different parts of *P. macrocarpa* fruit showed excellent antioxidant activities and good anti-inflammatory and high cytotoxic activities against HT-29, MCF-7, and HeLa cell lines [5]. Gallic acid isolated from *P. Macrocarpa* has been shown to possess anticancer properties against human esophageal cancer cells (TE-2) but no cytotoxic effect was observed on noncancerous (CHEK-1) cells [6]. The major compound of Phaleria obtained from *P. macrocarpa* fruits has been found to have high cytotoxic effect on MDA-MB231 breast cancer cell lines [7]. DLBS1425 of the extraction of *P. macrocarpa* flesh fruits inhibited proliferation of MDA-MB231 and MCF-7 cells, activation of caspase 9, and downregulation of Bax and Bcl-2 at the mRNA level [8]. In addition, it was also revealed that 2,6′,4-trihydroxy-4-methoxybenzophenone and 4′,6-dihydroxy-4-methoxybenzophenone 2-o*-β*-D-glucopyranoside isolated from the fruits displayed antiproliferation activity against the breast cancer cell line (MDA-MB231) and the formation of DNA fragments, as well as decreasing Bcl2 mRNA expression, increasing Bax mRNA expression and apoptosis inducer activity [9]. Furthermore, 2,6,4′-trihyroxy-4-methoxybenzophenone from an ethyl acetate extract of the leaves of *P. macrocarpa* possessed good antioxidant activity [10].

In our present study, the bioactive 2,6′,4-trihydroxy-4-methoxybenzophenone was isolated from the ethyl acetate extract of the *P. macrocarpa* fruits and analyzed using gas-chromatography mass spectrometry (GC-MS) and nuclear magnetic resonance, which were ^1^H, ^13^C, DEPT, HMQC, and HMBC. The HT-29 human colon cancer cell line was then selected for evaluation of bioactivity and molecular mechanisms to examine the efficiency of 2,6′,4-trihydroxy-4-methoxybenzophenone.

## 2. Materials and Methods

### 2.1. Extraction and Isolation of Bioactive Compounds

**Figure 1:**
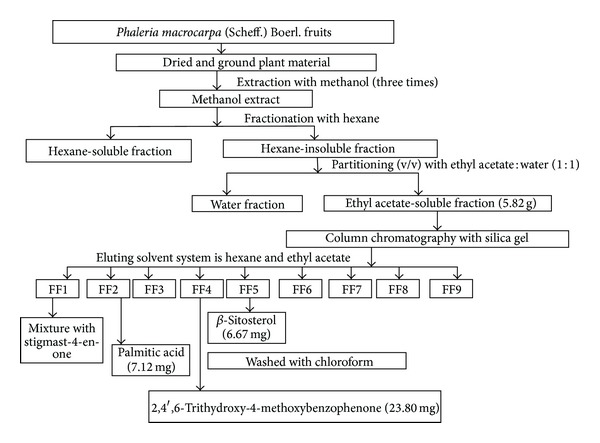
Extraction and isolation of bioactive compounds from ethyl acetate fraction of *Phaleria macrocarpa* (Scheff.) Boerl. fruits using silica gel column chromatography.

*P. macrocarpa *fruits were obtained from Yogyakarta, Indonesia (July 2009). The fruits (1 kg) were cut, washed, air-dried, and crushed. The powdered fruits were extracted with 70% methanol for three days at room temperature. The resulting solution was then filtered, combined, and concentrated under reduced pressure at 37°C using a rotary evaporator to yield a crude methanol extract. The methanol extract was then dissolved in hexane and this produced hexane soluble and insoluble fractions. The hexane soluble fraction was separated with water and an ethyl acetate solution (1 : 1) to yield water fractions and ethyl acetate fractions. The dried residue of the four extracts was then subjected to bioassays. The bioactive ethyl acetate fraction was subjected to column chromatography for isolation of bioactive compounds. The method for the extraction, fractions, and column chromatography of the fruits is summarized in Figure 1.

### 2.2. Analysis of Bioactive Compounds

#### 2.2.1. Gas Chromatography-Mass Spectrophotometry (GC-MS)

GC-MS was performed as previously described [11].

#### 2.2.2. Nuclear Magnetic Resonance (NMR)

The NMR analysis was carried out on a JEOL 400 MHz FT-NMR, (JEOL, Japan) with TMS (tetramethylsilane) as the internal standard. Spectra were obtained from Proton ^1^H-NMR (400 MHz), carbon ^13^C-NMR (400 MHz), HMBC, and HMQC analysis.

### 2.3. Preparation of Test Compound (2,4′,6-Trihydroxy-4-methoxybenzophenone)

The test compound used was 2,4′,6-trihydroxy-4-methoxybenzophenone isolated from *P.macrocarpa*. Stock solutions of 2,4′,6-trihydroxy-4-methoxybenzophenone were prepared at a concentration of 38 mM with absolute DMSO solution. The test sample was then prepared through serial dilution of the stock solution to give concentrations of 4 *μ*M, 38 *μ*M, 96 *μ*M, 192 *μ*M, and 384 *μ*M. The final concentration of DMSO in each treatment well was not in excess of 0.5% DMSO.

### 2.4. Cytotoxicity Screening for Bioactive Compounds

#### 2.4.1. Cell Culture

The HT-29 human colon carcinoma cell line, WRL-68 normal liver cells, and MRC-5 normal lung fibroblast carcinoma cells were purchased from the American Type Culture Collection (ATCC, USA). The cells were placed into tissue culture flasks and grown in a medium containing 10% fetal bovine serum, 100 *μ*g/mL of penicillin/streptomycin, and 100 *μ*g/mL of amphotericin B at 37°C in a humidified atmosphere containing 5% CO_2_.

#### 2.4.2. MTT Cell Proliferation Assay

The MTT (3,4,5-dimethylthiazol-2-yl)-2-5-diphenyltetrazolium bromide) cell proliferation assay was based on the protocol described by Mosmann [12]. Briefly, cells from a confluent tissue culture flask were spun at 1,000 rpm for five minutes and resuspended in 1.0 mL of growth medium. Cells were then seeded into 96 well plates and incubated in a CO_2_ incubator at 37°C for 24 hours to allow the cells to adhere and achieve 70%–80% confluence. After 24 hours, the media was removed and the test compound, at varying concentrations of 4, 38, 96, 192, and 384 *μ*M with 200 *μ*L of 10% medium, was added to the respective wells. For negative control, untreated cells were used. After 24 h, 48 h, and 72 h of incubation, respectively, each well had 10 *μ*L of the MTT stock solution added to the test sample and was then incubated in a dark place for three hours. The medium with the MTT solution was removed and 200 *μ*L of absolute DMSO was added to each well in order to solubilise the formazan crystals that had formed. The total amount of formazan crystals was examined by measuring the absorbance at 540 nm using an ELISA plate reader. The assay was carried out in triplicate.

The percentage of inhibition (%) was calculated using the following formula:

(1) percentage  of  inhibition  (%) =OD  control−OD  sample×100%  OD  control,

where OD = optical densities.

The IC_50_ value is the concentration of test compounds which cause 50% inhibition or cell death, average from three experiments, and was obtained by plotting the percentage of inhibition versus the concentration of test compounds [13].

### 2.5. Morphological Detection of Apoptosis

#### 2.5.1. Inverted and Phase-Contrast Microscopy

HT-29 cells were placed in a Petri dish (30 mm) at a density of 1 × 10^5^ cells/well plate and grown for 24 h. The test compound was then added at IC_50_ value and the cells were further incubated for 24 h, 48 h, and 72 h, respectively. After the various incubation periods, morphological changes in the apoptotic bodies of the HT-29 cells were examined by inverted and phase-contrast microscopy (*Leica*, Germany) and photographed.

#### 2.5.2. Fluorescence Microscopy

HT-29 cells were placed in a Petri dish (30 mm) at a density of 1 × 10^5^ cells/well plate and grown for 24 h. The test compound (115 *μ*M) was added to each dish and the cells were further incubated for 72 h. After the incubation period, the cells were detached with 0.25% accutase in phosphate buffer saline (PBS), the supernatant was discarded, and the cells were resuspended in 1 mL of PBS. Cells (100 *μ*L) were then incubated with 5 *μ*L of acridine orange (AO) and 5 *μ*L of propidium iodide (PI) for 10 min at room temperature in the dark. Stained cells (10 *μ*L) were put in three wells of the coated glass slides with 20 *μ*L of mounting media and stored at −20°C prior to analysis. These double stained cells were photographed using a fluorescence microscope (*Leica*, Germany). All experiments were performed in triplicate.

### 2.6. Annexin V Staining Assay

The quantification of cell death was determined by flow cytometry using the Annexin V-FITC apoptosis detection kit according to the manufacturer's instructions (BD Pharmingen, BD Bioscience, USA). Briefly, 1 × 10^6^ of the HT-29 cells were seeded into each Petri dish (30 mm) and after a 24 h incubation, various concentrations of the test compound were added and incubated for 24 h, 48 h, and 72 h, respectively. The cells were then washed with PBS, suspended in annexin V binding buffer and then added to an annexin V-FITC solution and propidium iodide (PI) for 10 minutes at room temperature. The samples were then analyzed using FACScalibur (BD Bioscience, USA) using CellQuest Pro analysis software (Becton Dickinson, USA).

### 2.7. Cell Cycle Analysis

HT-29 cells were placed in Petri dishes at a density of 1 × 10^5^ cells/well and grown for 24 h and then the cells were treated with 2,4′,6-trihydroxy-4-methoxybenzophenone (at IC_50_) at 37°C for 24 h, 48 h, and 72 h. After incubation, the cells were collected by trypsinization, fixed in ethanol, washed in warm PBS, and incubated at −20°C, overnight. Next, the cells were washed with 1 mL of cold PBS and stained with propidium iodide according to the Cycle TEST PLUS DNA Reagent Kit (Becton Dickinson, USA) protocol. The cells were sorted in a FACScalibur flow cytometer (BD, USA) using CellQuest Pro software (Becton Dickinson, USA) and a quantitative analysis of the cell cycle distribution was performed using a trial version of the ModFit LT software, version 4.0.

### 2.8. Western Blot Analysis

The HT-29 cells were cultured in 25 cm^3^ tissue culture flasks and incubated at 37°C under 5% CO_2_ until confluent. The cells were then treated with a double concentration of IC_50_ value for the 2,4′,6-trihydroxy-4-methoxybenzophenone for 24 h, 48 h, and 72 h, respectively. After incubation at the various times, the cells were detached with accutase in PBS, washed thoroughly with PBS twice, and were then lysed with protein lysis buffer (Sigma, USA) containingprotease inhibitors and shaken by hand for 15 min at room temperature. The lysates were then centrifuged at 13,000 ×g for 20 min at 4°C and the supernatant was collected for protein samples and kept at 40°C until SDS page analysis. Protein concentration was determined using a BCA (bicinchoninic acid) protein assay kit (Invitrogen, USA) with bovine serum albumin as the standard.

The proteins were transferred into polyvinylidene difluoride (PVDF) membranes by semidry transfer. Bands were visualized using the Western Max Horseradish Peroxidase Chromogenic Detection Kit protocol (AMRESCO, USA). Briefly, the membranes were blocked in a blocking buffer for 30 min at room temperature. Primary antibodies for Bcl-2, Bcl-xL, PUMA, Mcl-1, Bak, Bad, and Bax were then added. The membranes were incubated with gentle agitation at room temperature overnight. After incubation, the membranes were washed with washing buffer two times and then incubated with Western Max HRP Conjugated Anti-Rabbit Antibody for 45 min at room temperature with gentle agitation. After discarding the secondary antibody solution, the membranes were washed twice and incubated with DAB substrate solution containing hydrogen peroxide at room temperature until a brown color developed. Finally, the membranes were dried, photographed, and stored in the dark.

### 2.9. Data Analysis

The numbers of viable cells were counted using a haemocytometer with trypan blue exclusion. MTT test was performed in triplicate. The data of the cytotoxicity assay was expressed as the mean ± standard derivation (SD). The mean ± standard derivations (SD) in each cell cycle phase were also calculated for the annexin V-FITC/PI double staining assay. The results from treated and untreated control cells were analyzed using Student's *t*-test. Differences with a *P*  value of  >0.05 as determined using version 16.0 of SPSS were considered statistically significant.

## 3. Results and Discussions 

### 3.1. Extraction and Isolation

The yield of dark brown methanol extract (47.2 g, 4.7%), hexane fraction (2.10 g, 0.20%), ethyl acetate fraction (12.2 g, 1.22%), and water fraction (29.8 g, 2.90%) were determined. The ethyl acetate fraction (9.8 g) was subjected to silica gel column chromatography on a Merck Kieselgel 60 (400 g, 0.063–0.200 mm mesh size): initial elution with hexane, followed by ethyl acetate enriched with increasing percentages of acetone and monitored with TLC to give several main fractions: FF1 (0.7 g), FF2 (0.79 g), FF3 (0.42 g), FF4 (0.7 g), FF5 (0.5 g), FF6 (0.8), FF7 (1.5 g), FF8 (1.8 g), and FF9 (2.3 g). The components in these fractions were identified using GC-MS, NMR technique and some fractions were purified by recrystallization and separation with specific solvents.

GC-MS analysis of the ethyl acetate fraction of the *P. macrocarpa *showed the presence of phenol (*m*/*z* 94), 2,6-dimethoxy phenol (*m*/*z* 154), 2-methoxy phenol (*m*/*z* 124), *β*-sitosterol (*m*/*z* 414), stigmast-4-en-3-one (*m*/*z* 412), flamenol (*m*/*z* 140), palmitic acid (*m*/*z* 256), methyl palmitate, oleic acid (*m*/*z* 264), and other unknown components.

A mixture with stigmast-4-en-3-one (*m*/*z* 276) was detected in (FF 1), *β*-Sitosterol (*m*/*z* 414) was obtained from fraction 5 (FF 5), and palmitic acid was also obtained from fraction 2 (FF2) by identification GC-MS analysis. The pure bioactive compound was obtained from fraction 4 (FF 4) after recrystallization. This compound was identified as 2, 4′, 6-trihydroxy-4-methoxybenzophenone using NMR and GC-MS analysis.

### 3.2. Identification of Isolated Bioactive Compound 2,4′,6-Trihydroxy-4-methoxybenzophenone or 2,6-Dihydroxy-4-methoxyphenyl 4-Hydroxyphenyl methanone

**Figure 2:**
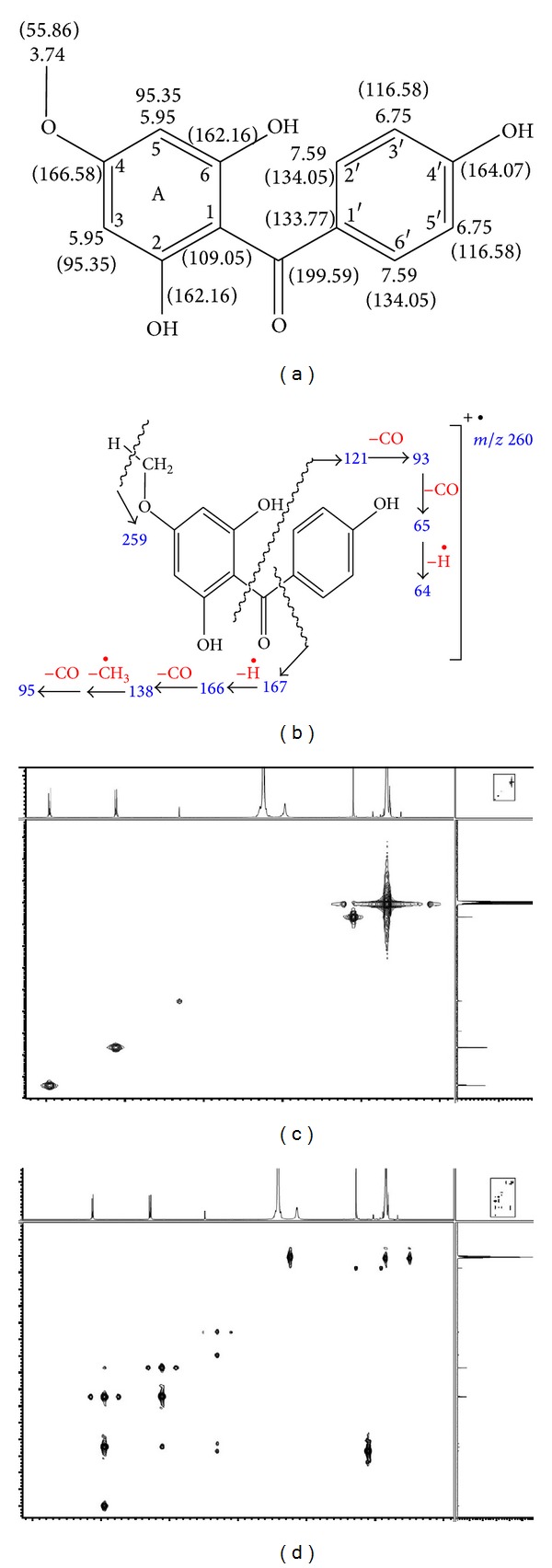
(a) Structure of 2,4′,6-trihydroxy-4-methoxybenzophenone showing assignment of protons and carbons. (b) Proposed fragmentation of 2,4′,6-trihydroxy-4-methoxybenzophenone. (c) HMBC analysis of isolated 2,4′,6-trihydroxy-4-methoxybenzophenone. (d) HMQC analysis of isolated 2,4′,6-trihydroxy-4-methoxybenzophenone.

**Figure 3:**
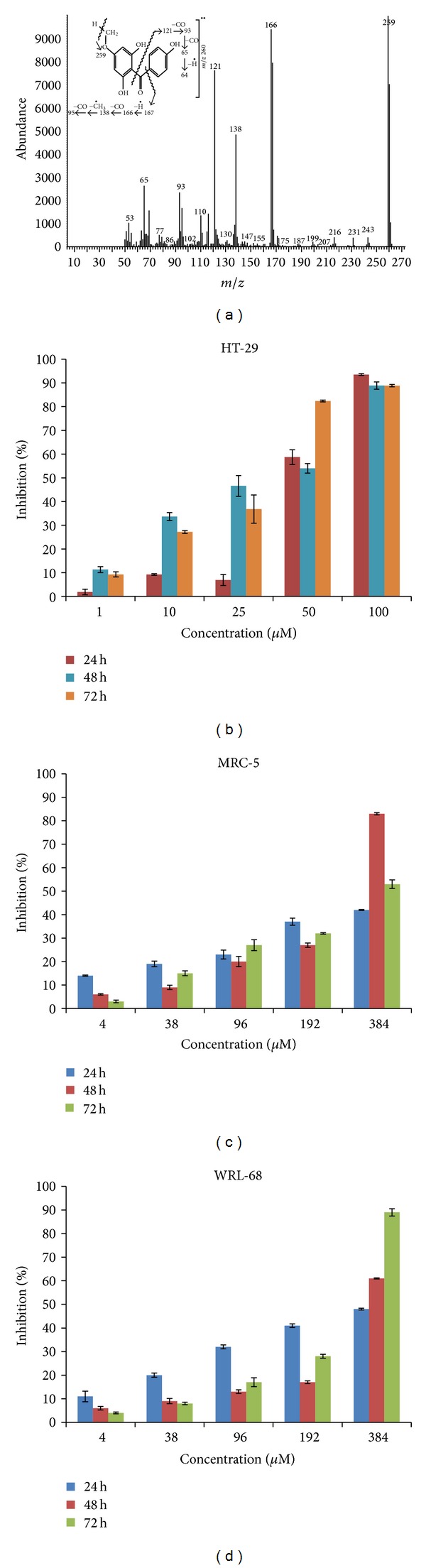
(a) Mass-spectrum of gas chromatography mass spectrometry (GC-MS) analysis of 2,4′,6-trihydroxy-4-methoxybenzophenone, (b) *In vitro*, cytotoxic effects of 2,4′,6-trihydroxy-4-methoxybenzophenone on (b) HT-29 colon cancer cells, (c) MRC-5 normal lung cells and (d) WRL-68 normal fibroblast cells. Cells were treated with various concentrations of 2,4,6′-trihydroxy-4-methoxybenzophenone from ethyl acetate fraction of *Phaleria macrocarpa *(Scheff.) Boerl. fruits for 24 h, 48 h, and 72 h prior to the determination of cytotoxicity by MTT cell proliferation assay. Each value is expressed as mean ± standard deviation of three measurements.

The structure of this compound was established on the basis of EI-MS data together with ^1^H and ^13^C NMR spectra as shown in Figure 2(a). The peak at retention time 43.93 min in the total ion chromatogram of fraction FF 4 gave a molecular ion peak at *m/z* 260 in the mass spectrum which was consistent with the molecular weight of 2,4′,6-trihydroxy-4-methoxybenzophenone having a molecular formula of C_14_H_12_O_5_. Fragment ions resulting from cleavages on either side of the carbonyl group were observed at *m/z* = 121 and 93, respectively, as shown in Figure 3(a). The formation of other fragment ions is explained in Figure 2(b).

The two pairs of equivalent aromatic hydrogen, namely, H_2′_/H_6′_ and H_3′_/H_5′_ in ring B and a pair of equivalent aromatic hydrogens in ring A, H_3_/H_5_, are visible at *δ* 7.59 (d), 6.75 (d), and 5.95 (s), respectively, in the ^1^H NMR spectrum (CDCl_3_/CD_3_OD, 400 MHz) (Table 1). The corresponding three equivalent pairs of aromatic carbons, C_2′_/C_6′_, C_3′_/C_5′_, and C_3_/C_5_, can also be seen at *δ* 134.05, 116.58, and 95.35 in the ^13^C NMR spectrum (CDCl_3_/CD_3_OD, 100 MHz) in Figure 2(a). The signals also showed an aromatic methoxy group at *δ* 3.74 and 55.86 in ^1^H and ^13^C NMR spectra, respectively. The ketone carbonyl signal appears at *δ* 199.59. Along with the chemical shifts of the remaining carbons, the assignment of all the NMR signals is summarized in Table 1 and Figure 2(a). The assignment of ^1^H and ^13^C NMR signals is also summarized together with the ^1^H-^13^C HMBC and HMQC data in Table 1 as well as in Figures 2(c) and 2(d).

### 3.3. Cytotoxicity Screening Using MTT Cell Proliferation Assay 

#### 3.3.1. Screening for Cytotoxic Activity of 2,4′,6-Trihydroxy-4-methoxybenzophenone

Cell proliferation is an important mechanism for the growth, development, and regeneration of eukaryotic organisms but it is, nevertheless, also the primary cause of some of the most debilitating diseases, one of which is cancer [14].

**Figure 4:**
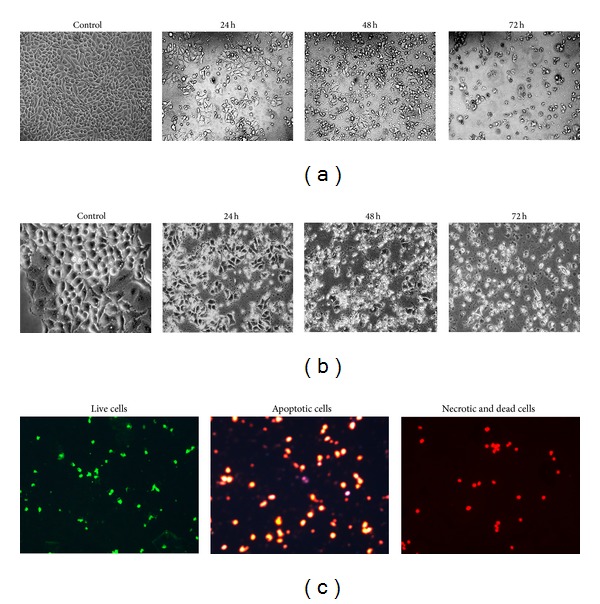
(a) Cells were treated with compound (30 µM) for 24 h, 48 h, and 72 h which induced morphological changes typical of apoptosis in HT-29 colon carcinoma cells. Control or treated cells were observed under inverted microscope and photographed. (b) Control or treated cells were observed under phase contrast microscope and photographed. (c) Treatment with IC_50_ value (30 µM) of 2,4′,6-trihydroxy-4-methoxybenzophenone for 48 h induces morphological changes typical of apoptosis in HT-29 colon cancer cells. After being stained with acridine orange and propidium iodide, treated cells were observed under fluorescence microscopy—live cells stained (green colour), apoptotic cells (orange), and necrotic cells or dead cells (red).

In this study, the cytotoxicity screening of the 2,4′,6-trihydroxy-4-methoxybenzophenone against the HT-29 human colon carcinoma cell line was performed using the MTT cell proliferation assay and the cell viability of HT-29 cells was measured using the trypan blue exclusion assay. The HT-29 cells were treated with various concentrations (4, 38, 96, 192, and 384 *μ*M) for 24 h, 48 h, and 72 h, respectively. The results showed that inhibition of cell growth significantly increased in a dose-dependent and time-dependent manner when the HT-29 cells were treated with 2,4′,6-trihydroxy-4-methoxybenzophenone at 4, 38, 96, 192, and 384 *μ*M for 24 h, 48 h, and 72 h except for 96 *μ*M for 24 h as shown in Figures 3(b) and 4(a). The IC_50_ values of the compound against the HT-29 cells were 172 ± 2.21, 144 ± 2.66, and 122 ± 1.69 *μ*M for 24 h, 48 h, and 72 h. In contrast, the 2,4′,6-trihydroxy-4-methoxybenzophenone compound displayed mild cytotoxic effect on human normal liver cells (WRL-68) with IC_50_ values of ≥384 ± 1.02, 331 ± 0.69, and 235 ± 1.03 *μ*M for 24 h, 48 h, and 72 h, respectively, as shown in Figure 3(d) and human normal fibroblast lung cells (MRC-5) with IC_50_ values of  ≥384 ± 1.00, 269 ± 0.96, and 356 ± 1.21 *μ*M for 24 h, 48 h, and 72 h, respectively, as shown in Figure 3(c).

### 3.4. Morphological Studies

HT-29 cells were treated with the isolated bioactive compound at IC_50_ concentrations (115 *μ*M) for incubation periods of 24 h, 48 h, and 72 h, respectively. After these incubation periods, the morphological changes in the cells were examined using inverted and phase-contrast microscopy. The cells displayed changes that are known to be associated to apoptosis including membrane blebbing, cell shrinkage, chromatin condensation, apoptotic nuclei, and DNA fragmentation, (Figures 4(a) and 4(b)).

### 3.5. Fluorescence Microscopy

The cells were observed under a fluorescence microscope at different excitations after staining with acridine orange and propidium iodide (AO/PI) for nuclei containing DNA. The results showed that the viable cells revealed the green nuclei, dead cells and late apoptotic and necrotic cells displayed red nuclei, and early apoptotic cells indicated orange nuclei in Figure 4(c). Thus, AO/PI staining of HT-29 showed that the cells had undergone remarkable morphological changes in apoptotic bodies.

### 3.6. Annexin V Staining Assay

The most important molecular mechanism used in the treatment of anticancer drugs is programmed cell death or apoptosis in the chemotherapeutic approach [15, 16]. In our study, staining with Annexin V-FITC and propidium iodide (PI) may distinguish between intact cells, early apoptosis, late apoptosis or cell death [17]. We also used single staining with Annexin V-FITC or PI to distinguish between live cells and dead cells; however, single staining could not distinguish between early apoptotic cells, late apoptotic cells or dead cells, or necrotic and live cells.

Cells were treated with various concentrations (96 *μ*M, 192 *μ*M, and 288 *μ*M) of the compound for 24 h, 48 h, and 72 h, respectively. They were then harvested with accutase and phosphate buffer saline and centrifuged, after which they were stained with Annexin V-FITC and propidium iodide and analyzed in FACScalibur with CellQuest Pro software. The number of early apoptotic cells, late apoptotic cells, live cells, and necrotic cells was counted in every 10,000 cells of each treatment [18]. All data was expressed as the mean ± SD (standard deviation). The standard deviation was calculated for the treated and untreated cells. The SPSS program (version 16.0) found a significant difference between the treated and untreated cells (*P* > 0.05).

**Figure 5:**
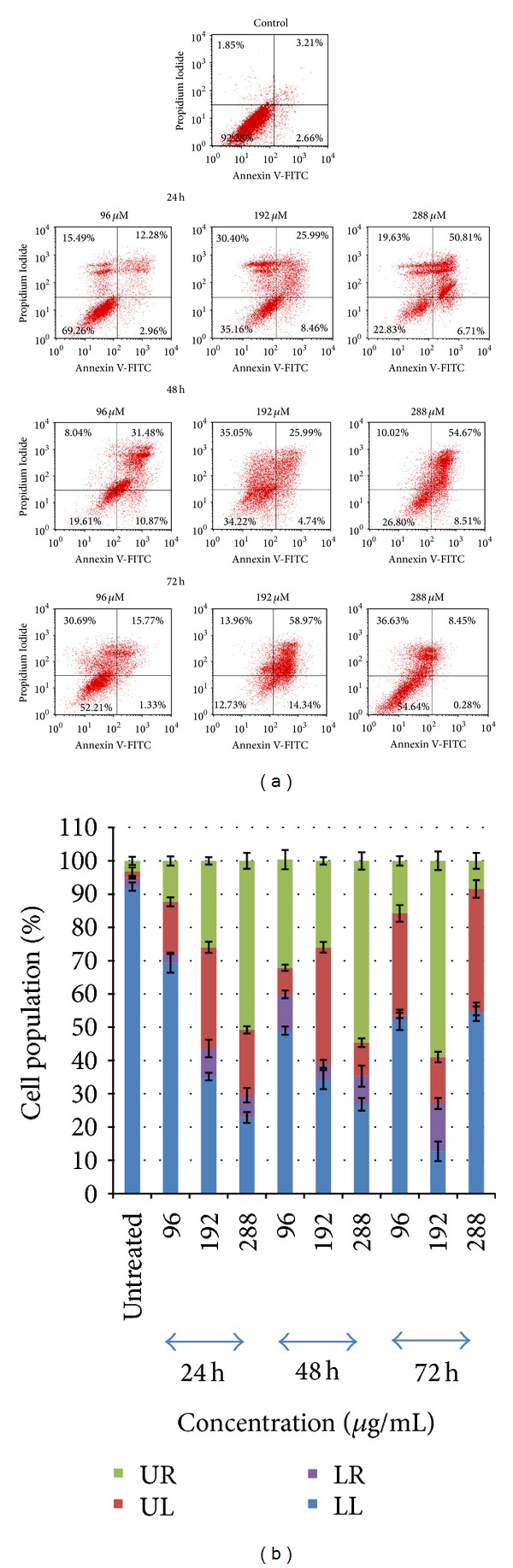
(a) Effects of 2,4′,6-trihydroxy-4-methoxybenzophenone on induction of apoptosis in HT-29 cells. The cells were treated with different concentrations of compound (25 µM, 50 µM, and 75 µM) in a time-dependent manner (24 h, 48 h, and 72 h), labelled with FITC annexin V and PI. Viable cells = LL; Early apoptotic cells = LR; late or secondary necrotic cells = UR; primary necrotic cells = UL. (b) Histogram representation of the quantitative percentage of viable cells (LL), early apoptotic cells (LR), primary necrotic cells (UL), and late apoptotic cells or secondary necrotic cells (UR) of HT-29 treatment with different concentration of 2,4′,6-trihydroxy-4-methoxybenzophenone for 24 h, 48 h, and 72 h.

In flow cytometry analysis of Annexin V-FITC/PI double staining, the late or secondary necrotic apoptotic cells were visible in the upper right (UR) and early apoptotic cells in the lower right (LR) quadrants, while the primary necrotic cells were visible in the upper left (UL) and live cells in the lower left (LL) quadrants, respectively. In untreated HT-29 cells, 92.28% of the cells were viable, 1.85% was in early apoptosis and 3.21% cells were in late apoptosis, or secondary necrotic stage. When the HT-29 cells were treated with 96 *μ*M, 192 *μ*M, and 288 *μ*M of the compound for 24 h, the viable cells were 69.26%, 35.16%, and 23.83%, the primary necrotic cells were 15.49%, 30.40%, and 19.63%, the early apoptotic cells were 2.96%, 8.46%, and 6.71%, and the late apoptotic cells were 12.28%, 25.98%, and 50.81%, respectively. After 48 h incubation, live cells were at 49.61%, 34.22%, and 26.80%, primary necrotic cells were at 8.04%, 35.05%, and 10.02%, early apoptotic cells were at 10.87%, 4.74%, and 8.51%, and late apoptotic cells were at 31.48%, 25.55%, and 54.67%, respectively. After 72 h incubation, live cells were at 52.21%, 12.73%, and 54.64%, primary necrotic cells were at 30.69%, 13.96% and 36.63%, early apoptotic cells were at 1.33%, 14.34% and 0.28%, and late apoptotic or secondary necrotic cells were at 15.77%, 58.97%, and 8.45%, respectively. All these data are shown in Figures 5(a) and 5(b).

The results indicated that the effects of programmed cell death or apoptosis on HT-29 colon carcinoma cells were induced by 2,4′,6-trihydroxy-4-methoxybenzophenone in a time-and dose-dependent manner.

### 3.7. Cell Cycle Analysis

One of the most important mechanisms in anticancer drug treatment is cell cycle arrest, which can be measured by DNA content. DNA (deoxyribonucleic acid) plays an essential role in cell reproduction and cell life and death and also carries genetic information for all living organisms and consists of two sets of chromosomes [15, 16]. When 2N DNA is present in the G_0_ and G_1_ phases of each cell cycle, the G_2_ and M phases in the cell cycle are presented by 4N DNA which has a double 2N number of chromosomes while the S-phase synthesizes DNA replication using flow cytometry analysis.

**Figure 6:**
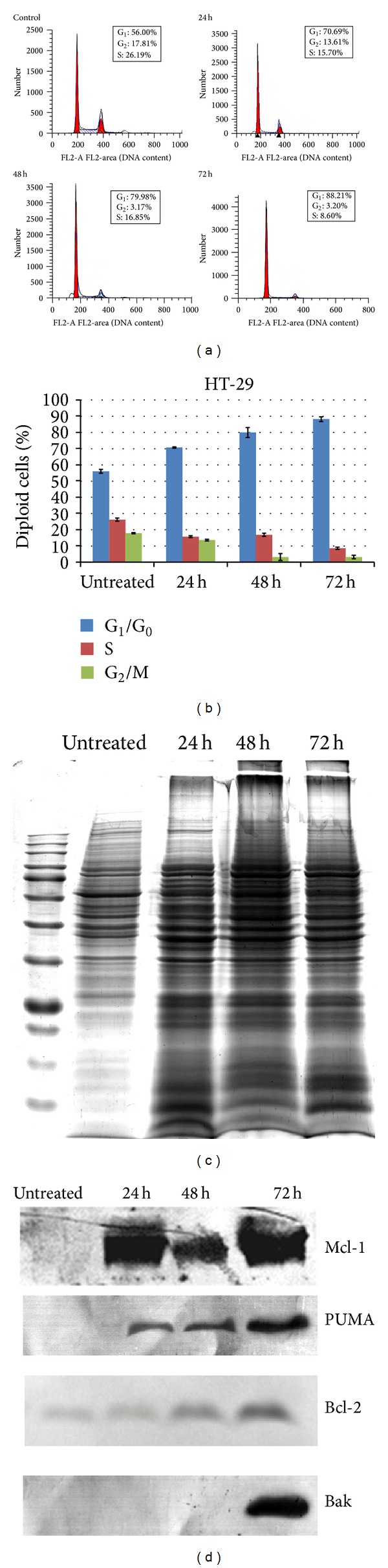
(a) Effect of 2,4′,6-trihydroxy-4-methoxybenzophenone on HT-29 cell cycle. Cells were treated with the IC_50_ value (30 µM) concentrations of compound for 24 h, 48 h, and 72 h and analyzed by flow cytometry after staining with PI. Percentages of diploid cells (DNA content) at G_0_/G_1_, S, and G_2_/M phases of HT-29 cells were determined after 24 h, 48 h, and 72 h incubation periods. (b) Histogram showing quantitative percentage of diploid cells (DNA content) in each cell cycle phase without treatment and with treatment. (c) The expression of the whole protein in Coomassie blue staining after compound induced apoptosis in HT-29 cells. (d) The expression of antiapoptotic protein and proapoptotic protein in western blotting analysis.

The HT-29 cells were treated with isolated bioactive 2,4′,6-trihydroxy-4-methoxybenzophenone for 24 h, 48 h, and 72 h. Treated and untreated cells were harvested with accutase and phosphate buffer saline, centrifuged, and stained with propidium iodide. The HT-29 cells were then analyzed by flow cytometry using CellQuest Pro software and their distribution in different phases of the cell cycle is shown in Figure 6(a). The resulting data were tabulated using the trial version of the ModFit L.V 4.0 software to represent a percentage of each cell cycle phase (G_0_/G_1_, S, and G_2_/M), as shown in Figure 6(b).

Treatment with the 2,4′,6-trihydroxy-4-methoxybenzophenone compound (at IC_50_ value of 115 *μ*M) resulted in cell cycle arrest in the G_0_/G_1_ phase in a time-dependent manner. Standard deviation was calculated for the treated and untreated cells. The significance of the difference between the treated and untreated cells in each cell cycle phase, namely, G_0_/G_1_, S and G_2_/M, was determined by Student's *t*-test and the *P*  value was >0.05 using SPSS, version 16.0.

After incubation for 24 h, 48 h, and 72 h, the percentage of diploid cells in the G_0_/G_1_ phase progressively increased to 70.69%, 79.98%, and 88.21% and the cell percentage in the G_2_/M and S phases was 13.61%, 3.17%, and 3.20% and 15.70%, 16.85%, and 8.60%, respectively (Figure 6(b)). The aneuploid cells in the G_0_/G_1_ and S phases were not stable because of abnormal chromosome problems in the centrioles where extra or missing chromosomes occurred.

Every diploid cell has two sets of chromosomes (2N) which occur in the G_0_/G_1_ phase of each cell cycle and tetraploid cells have double the normal 2N number of chromosomes (4N), which occur in the G_2_/M phase of each cell cycle; however, aneuploidy refers to an abnormal number of chromosomes, which was observed as a significant difference between the G_0_/G_1_ and G_2_/M phases in the flow cytometry analysis. We observed that the number of diploid cells and tetraploid cells in the G_0_/G_1_ and S phases of each cell cycle were heavily reliant on one another.

Thus, we have shown here that 2,4′,6-trihydroxy-4-methoxybenzophenone at 115 *μ*M induced apoptosis in HT-29 cells during G_0_/G_1_ arrest and the inhibition of cell growth could be a result of the induction of apoptosis, which may be resolved by cell cycle arrest, which may consequently result in programmed cell death.

### 3.8. Western Blot Analysis

Apoptosis or programmed cell death consists of three major pathways: the intrinsic pathway, the extrinsic or death-receptor pathway, and the signalling pathways that indicates death-associated proteolytic or nucleolytic activities.

The Bcl-2 family of proteins play an essential role in regulating the mitochondria-dependent pathway of apoptosis for health and diseases [19]. Bcl-2 proteins are alternative targets for both pro- and antiapoptotic therapeutic approaches [20]. There are two groups of proteins involved in apoptosis—antiapoptotic or prosurvival proteins, such as Bcl-2, Bcl-xl Bcl-w, Mcl-1, and A1 and proapoptotic proteins such as Bax, Bak, PUMA, Bok, Bad, Bid, Bik, Blk, Hrk, BNIP3, and BimL. The Bcl-2 family of proteins have been shown to regulate apoptosis in response to chemotherapy, both *in vivo* and *in vitro* [21, 22]. The Bcl-2 family of proteins are located in the endoplasmic reticulum membrane, the nuclear envelope, and in the mitochondrion's outer membranes [23]. The Bcl-2 prosurvival proteins directly or indirectly regulate the release of cytochrome c from mitochondria.

A member of the BH3 subgroup of the Bcl-2 family of proteins is PUMA (p53-upregulated mediator of apoptosis), which interacts with antiapoptotic Bcl-2 family proteins such as Bcl-xL, Bcl-2, Mcl-1, Bcl-w, and A1 or proapoptotic proteins such as Bax and Bak. It also involves p53-dependent and p53-independent apoptosis induced by different signalling pathways. Another antiapoptotic protein from the Bcl-2 family is the myeloid cell leukemia sequence 1 (Mcl-1), which is involved in the upregulation of apoptosis and survival regulation, and also plays a part in melanoma cell resistance towards Fas-mediated apoptosis. Inhibiting the mitochondrial signalling pathway of apoptosis can directly affect cancer therapy. It is well known that Bcl-2 is a potent antiapoptotic protein which is expressed in HT-29 cells. In our study, the expression of apoptosis and cell cycle associated proteins was determined using western blotting after the HT-29 cells were treated with 2,4′,6-trihydroxy-4-methoxybenzophenone. The expression of antiapoptotic (Bcl-2 and Mcl-1) and proapoptotic protein (PUMA and Bak) in HT-29 colon human adenocarcinoma cells line was examined using the western blot technique. To clarify the mechanisms by which the compound induced apoptosis of HT-29 cells, the cells were incubated at an IC_50_ concentration (115 *μ*M) of 4′,6-trihydroxy-4-methoxybenzophenone for 24 h, 48 h, and 72 h. Proteins were then extracted for western blotting. As is shown in Figures 6(c) and 6(d), the expression of Mcl-1 and PUMA was significantly upregulated when the HT-29 cells were treated with the compound for 24 h, 48 h and 72 h. The expression of Bcl-2 and Bak was only up-regulated for 72 h treatment of HT-29 cells. In apoptosis-related protein expression after treatment with the compound in HT-29 cells, the upregulation of Bcl-2 was found to be in a time-dependent manner. Our results showed that the G_0_/G_1_ arrest and apoptosis of the 2,4′,6-trihydroxy-4-methoxybenzophenone in HT-29 cells might be associated with the upregulation of the anti-apopptotic proteins Bcl-2 and Mcl-1, as well as the pro-apototic proteins Bak and PUMA. Recent researchers also demonstrated that DLBS1425 showed downregulation of Bax and Bcl-2 which are COX-2, cPLA2, and VEGF-C and c-fos and HER-2/neu mRNA expression in MDA-MB231 cells [8]. Using western blotting, we showed that Bcl-2, Mcl-1, and Bak and PUMA regulation may be involved in compound-induced apoptosis. Thus, we have shown that the Bcl-2 family of proteins may be directly or indirectly involved in the apoptotic effect of the compound in HT-29 colon human carcinoma cell lines and that the 2,4′,6-trihydroxy-4-methoxybenzophenone may induce apoptosis in HT-29 cells through pathways involving Bcl-2.

## 4. Conclusion

Our study showed that 2,4′,6-trihydroxy-4-methoxybenzophenone inhibits cell proliferation and is able to induce apoptosis in HT-29 cells. Western blot analysis suggested that the compound induced apoptosis by upregulating Bcl-2, PUMA, Mcl-1, and Bak proteins. These data suggested that the mechanism of cell cycle arrest in the G_0_/G_1_ phase may be related to Bcl-2 proteins and play a vital role in regulating the mitochondria-dependent pathway of apoptosis.

## Figures and Tables

**Table 1:** ^
1^H NMR (CDCl_3_/CD_3_OD, 400 MHz), ^13^C NMR (CDCl_3_/CD_3_OD, 100 MHz), HMQC, and HMBC data of isolated 2,4′,6-trihydroxy-4-methoxybenzophenone or 2,6-dihydroxy-4-methoxyphenyl 4-hydroxyphenyl methanone.

Position/group	*δ*C	*δ*H (mult, *J* in Hz)	^ 1^H-^13^C HMBC	HMQC
1	109.05	—	—	—
2/6	162.16	—	—	—
3/5	95.35	5.95 (*s*)	C-1, C-2/C-6, C-4	C-3/C-5
4	166.58	—	—	—
1′	133.77	—	—	—
2′/6′	134.05	7.59 (*d*, 8.8)	C-1′, C-3′/C-5′, C-4′, C=O	C-2′/C-6′
3′/5′	116.58	6.75 (*d*, 8.8)	C-1′, C-2′/C-6′, C-4′	C-3′/C-5′
4′	164.07	—	—	—
C=O	199.59	—	—	—
OCH_3_	55.86	3.74 (*s*)	C-4	COCH_3_

## Abbreviations

g: Gram
mL: Milliliter
min: Minutes
h: Hours
*m*/*z*: Molecular weight
AO/PI: Acridine orange and propidium iodide.
